# Reorienting catch-up growth research toward early-life prevention of metabolic disorders using natural products

**DOI:** 10.3389/fendo.2026.1800979

**Published:** 2026-05-28

**Authors:** Hendra Stevani, Habibie Habibie, Asbah Asbah, Firzan Nainu

**Affiliations:** 1 Doctoral Program in Pharmacy, Faculty of Pharmacy, Hasanuddin University, Tamalanrea, Makassar, Indonesia; 2 Program Study of Bachelor of Applied Pharmacy, Department of Pharmacy, Health Polytechnic of Makassar, Ministry of Health of the Republic of Indonesia, Baji Gau, Makassar, Indonesia; 3 Department of Pharmacy, Faculty of Pharmacy, Hasanuddin University, Tamalanrea, Makassar, Indonesia; 4 Unhas Fly Research Group, Faculty of Pharmacy, Hasanuddin University, Tamalanrea, Makassar, Indonesia

**Keywords:** catch-up growth, diabetes, early-life intervention, metabolic reprogramming, natural products, stunting

## Introduction

Stunting remains a major public health concern in many low- and middle-income countries where dietary quality, food security, and socioeconomic stability are constrained (1). Catch-up growth is commonly interpreted as a marker of recovery; however, excessive post-stunting growth has been associated with adverse metabolic outcomes including insulin resistance (2), central obesity (3), type 2 diabetes (4), mitochondrial dysfunction, and oxidative stress (5). Rapid catch-up growth has also been linked to adipocyte hyperplasia, which may increase long-term cardiometabolic risk (6). These findings highlight limitations of nutritional strategies that prioritize weight gain without addressing metabolic quality.

Early life represents a critical window for prevention because metabolic programming remains highly plastic during infancy and childhood (7). During catch-up growth, rapid nutritional rehabilitation may interact with pre-existing metabolic vulnerability and predispose individuals to long-term metabolic dysfunction. Mechanisms including dysregulated insulin-IGF signaling, mitochondrial dysfunction, and oxidative stress are increasingly recognized (3), yet research specifically addressing early-life prevention of metabolic disorders associated with catch-up growth remains limited.

Natural products and plant-derived bioactive compounds represent promising candidates for such preventive strategies. Many exhibit antioxidant, anti-inflammatory, insulin-sensitizing, and mitochondrial-protective effects targeting pathways implicated in catch-up growth-associated metabolic dysfunction. Their relative safety, affordability, and cultural acceptability support their potential relevance for early-life interventions, and emerging evidence suggests that early exposure to specific bioactive compounds may influence metabolic programming and reduce later metabolic disease risk (8).

Accordingly, this opinion argues that catch-up growth should be reconsidered not only as a marker of anthropometric recovery but also as a metabolically sensitive period for preventive intervention. Reorienting research toward early-life metabolic prevention-particularly through safe and accessible natural bioactive compounds-may help mitigate the long-term metabolic consequences of childhood stunting. In this article, metabolic programming refers to long-term metabolic changes induced by early-life environmental exposures, metabolic plasticity describes adaptive metabolic capacity during developmental windows, and metabolic vulnerability indicates increased susceptibility to metabolic dysregulation.

## Conceptual framework: maladaptive metabolic plasticity during catch-up growth

Catch-up growth following early-life undernutrition induces heightened metabolic plasticity (9). While adaptive, rapid growth in a vulnerable context may shift toward maladaptive metabolic plasticity, characterized by dysregulated insulin–IGF signaling, mitochondrial dysfunction, oxidative stress, and low-grade inflammation (10), contributing to adipocyte expansion and increased cardiometabolic risk (11).

We propose this phase as a critical window for metabolic modulation using bioactive compounds, such as polyphenols and flavonoids, which target oxidative, inflammatory, and insulin signaling pathways. This reframes catch-up growth as a metabolically sensitive period where trajectories may be redirected.

This concept can be operationalized using primary indicators, including insulin sensitivity (e.g., HOMA-IR) and core metabolomic signatures, reflecting systemic metabolic regulation, and supportive indicators, including oxidative stress markers (ROS, MDA), inflammatory cytokines, and mitochondrial function, capturing underlying cellular dysfunction. Together, these measures reflect impaired insulin signaling, elevated stress responses, and altered energy metabolism characteristic of maladaptive metabolic plasticity.

## Catch-up growth and long-term metabolic risk

Evidence linking catch-up growth with long-term metabolic risk provides important context for this framework. Early postnatal growth plays a key role in shaping later metabolic health (9). Plasma metabolite profiles at three months of age are associated with body composition at two years (12), a period that often coincides with the onset of catch-up growth in children with a history of stunting. Metabolomic evidence suggests that early metabolic alterations associated with growth restriction and catch-up growth involve disruptions in lipid signaling, amino acid metabolism, and inflammatory pathways (12–14). These alterations include changes in circulating metabolites detectable across early-life biological compartments (13–15). Specific biomarkers, such as myo-inositol, have been linked to altered glucose metabolism in growth-restricted neonates (16). Collectively, these observations support the view that catch-up growth occurs within a metabolically altered and vulnerable physiological context.

Although rapid catch-up growth is frequently linked to increased cardiometabolic risk, the evidence is not entirely uniform. Some longitudinal studies indicate that moderate catch-up growth may improve survival and neurodevelopment in undernourished populations. However, excessive or rapid catch-up growth-particularly when characterized by disproportionate fat accumulation-has consistently been associated with insulin resistance and later metabolic dysfunction (17). Moreover, much of the mechanistic evidence derives from experimental animal models, while metabolic outcomes in humans are influenced by complex environmental and nutritional factors. Together, these observations suggest that accelerated catch-up growth may amplify pre-existing metabolic vulnerability, consistent with the concept of maladaptive metabolic plasticity.

## Early life as a critical window for metabolic programming

Early life represents a critical period during which metabolic programming remains highly plastic and responsive to environmental and nutritional conditions. Recent advances indicate that early-life metabolic programming is shaped not only by nutrient availability but also by regulatory systems such as the gut microbiome and epigenetic mechanisms. The early-life gut microbiome modulates energy balance, inflammatory responses, and insulin sensitivity, while epigenetic processes-including DNA methylation and histone modification-mediate long-term metabolic effects of early nutritional exposures (18, 19).

In children with early growth restriction, reduced insulin signaling capacity may develop as an adaptive energy-conserving response, as described in the thrifty phenotype hypothesis (20). During rapid catch-up growth, increased caloric intake stimulates insulin secretion to support accelerated tissue growth; however, persistent hyperinsulinemia may promote insulin receptor desensitization and impaired downstream signaling, ultimately contributing to insulin resistance.

Rapid catch-up growth-particularly in the context of high fat and carbohydrate intake-often favors visceral fat accumulation rather than lean mass accretion (21). These effects may be reinforced by epigenetic modifications established during fetal undernutrition, including altered methylation of genes involved in insulin signaling such as IRS-1 and PI3K, thereby promoting lipid accumulation and metabolic dysfunction.

**Figure 1:**
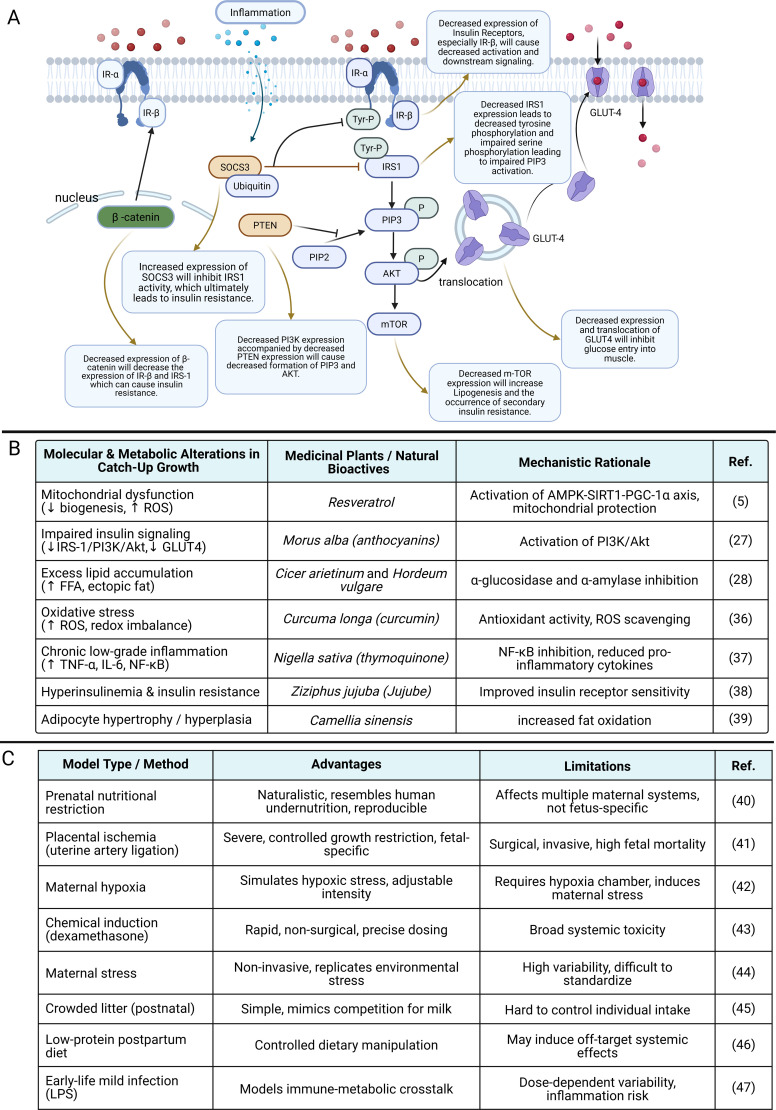
Conceptual framework of insulin resistance during catch-up growth and its potential modulation by natural bioactives. **(A)** Key pathways involved in impaired insulin signaling under inflammatory and oxidative stress conditions, illustrated as a conceptual representation of converging mechanisms. **(B)** Selected bioactive compounds and their mechanistic targets relevant to catch-up growth-associated metabolic disturbances, presented as representative examples rather than a comprehensive listing. **(C)** Common experimental models used to study catch-up growth and metabolic programming, highlighting key advantages and limitations as illustrative research approaches. Created in BioRender. Nainu, F. (2026) https://BioRender.com/ecyxkti.

Oxidative stress and inflammatory signaling play a central role in disrupting insulin signaling through impairment of key metabolic pathways (22). In parallel, mitochondrial dysfunction during rapid catch-up growth increases reactive oxygen species (ROS) production, further amplifying inflammatory responses and reinforcing insulin resistance pathways (23). These interconnected processes, summarized in **Figure 1A**, support the concept that catch-up growth promotes insulin resistance through the convergence of inflammation, oxidative stress, and impaired insulin signaling.

## Early life prevention of metabolic disorders: evidence, relevance to catch-up growth, and research readiness

A growing body of evidence indicates that many metabolic disorders traditionally diagnosed in adulthood originate from early-life disturbances in metabolic programming. Interventions during infancy and childhood may reduce long-term cardiometabolic risk by targeting pathways that remain plastic during early development (23). This preventive perspective is particularly relevant in catch-up growth, where rapid nutritional rehabilitation intersects with metabolic vulnerability (24).

The biological mechanisms linking catch-up growth to later metabolic disease-including impaired insulin signaling, oxidative stress, inflammation, and mitochondrial dysfunction-are increasingly recognized. These pathways overlap with targets of established metabolic interventions, supporting the plausibility of early preventive strategies.

Research capacity to investigate early-life prevention is also emerging. Validated animal models of stunting and catch-up growth enable controlled evaluation of early interventions, while advances in metabolomics and molecular profiling provide sensitive biomarkers for assessing metabolic programming and intervention responses (**Figure 1C**). However, translation into effective preventive strategies will require carefully designed studies. Overall, current evidence suggests that catch-up growth represents a tractable target for early-life metabolic prevention.

## Discussion

### Natural bioactive compounds as metabolic modulators

Plant-derived bioactive compounds have long been explored for their capacity to influence pathways relevant to cardiometabolic disease. Antioxidants such as alpha-lipoic acid improve insulin sensitivity and reduce oxidative stress in diabetic conditions (25), while flavonoids, polyphenols, and omega-3 fatty acids modulate insulin signaling, inflammation, and lipid metabolism (26). In the context of catch-up growth, their relevance lies in modulating key pathways-oxidative stress, inflammation, mitochondrial function, and insulin signaling-that are already perturbed during early-life metabolic adaptation. This overlap suggests that metabolic quality, rather than caloric adequacy alone, may influence long-term outcomes following nutritional rehabilitation.

From a developmental perspective, catch-up growth represents a period of heightened metabolic plasticity during which these pathways remain modifiable, raising the possibility that targeted metabolic modulation could influence long-term trajectories. For example, anthocyanins have been shown to activate PI3K/Akt signaling (27), while resveratrol improves mitochondrial function and antioxidant capacity in experimental models of catch-up growth (5). These findings illustrate how bioactive compounds interact with core regulatory networks implicated in maladaptive metabolic programming. Selected bioactive compounds and their mechanistic targets relevant to catch-up growth-associated metabolic disturbances are illustrated in **Figure 1B** as representative examples rather than as an exhaustive list, and are supported by previous studies (28–39).

Experimental evidence supports this conceptual link. Plant-derived compounds, including anthocyanins and legume-derived bioactives, have been shown to enhance insulin sensitivity, regulate key signaling pathways, and modulate metabolic homeostasis in relevant models (27, 40). These findings should be interpreted as mechanistic support rather than direct clinical evidence.

Emerging data from in silico and preclinical studies suggest that multiple bioactive agents may converge on shared metabolic targets, including insulin signaling and mitochondrial regulation (40). This raises the possibility that combination or low-dose strategies may achieve metabolic effects while minimizing safety concerns in early-life contexts (41).

However, translation into clinical practice remains constrained. Current pediatric guidelines do not support pharmacological supplementation during rapid nutritional rehabilitation, and most available evidence derives from experimental models or adult populations. Accordingly, these compounds may be better positioned within food-based or low-dose nutritional strategies. Further research is needed to define optimal dosing, timing, and safety in children undergoing catch-up growth.

### Translational evidence from human studies

Evidence supporting the metabolic effects of plant-derived bioactive compounds extends beyond experimental models to include randomized clinical trials and population-based studies, although most evidence derives from adult populations. Human studies report beneficial metabolic effects of polyphenol-rich diets and individual bioactive compounds. Randomized clinical trials show that resveratrol supplementation improves insulin sensitivity and reduces oxidative stress in individuals with metabolic disorders (42), while large intervention studies such as the PREDIMED trial demonstrate reduced cardiovascular risk with polyphenol-rich diets (43). Longitudinal cohort studies further indicate that early-life nutritional exposures influence long-term cardiometabolic health (44). However, these studies are largely conducted in adults and not designed to evaluate early-life interventions.

Additional support for early-life metabolic prevention comes from nutritional studies. Breastfeeding has been associated with reduced risk of obesity and type 2 diabetes later in life (45), and randomized trials modifying protein content in infant formula demonstrate that early nutritional exposures influence long-term adiposity (46). However, direct clinical evidence that specific bioactive compounds administered during early life prevent adult metabolic disease remains limited, highlighting the need for carefully designed studies targeting catch-up growth.

### Limitations and research gaps

Several limitations of the current evidence base must be acknowledged. The chemical complexity of plant-derived compounds presents challenges for standardization and reproducibility, while variability in phytochemical composition across plant sources and extraction methods complicates consistent bioactive profiles. Dose-response relationships for many compounds remain incompletely characterized, particularly in pediatric populations, and differences in bioavailability and metabolism complicate translation from animal models to humans. Long-term safety data for early-life exposure remain limited, and rigorous clinical validation is required before application in pediatric populations.

No pharmacological therapy is currently recommended to prevent metabolic disorders in children undergoing rapid nutritional rehabilitation, as emphasized by WHO and ESPGHAN pediatric nutrition guidelines (47). Therefore, interventions during this period must prioritize safety, feasibility, and cultural acceptability. Prevention trials in early-life populations also present ethical and methodological challenges, including long follow-up periods and safety considerations. Accordingly, natural bioactive compounds should be viewed not as stand-alone therapeutics but as metabolic modulators that support healthier metabolic adaptation during catch-up growth.

## Conclusion

Although catch-up growth is essential for restoring linear growth in previously stunted children, it may also increase long-term metabolic risk if metabolic quality is not addressed. Reframing catch-up growth as a window for early-life metabolic prevention provides a clear direction for reducing the life-course burden of metabolic disease. Future studies should test whether early-life metabolic modulation using bioactive compounds during catch-up growth can normalize dysregulated pathways and reduce long-term cardiometabolic risk.

A stepwise translational approach is warranted, progressing from mechanistic and preclinical studies to longitudinal cohort studies, and ultimately to carefully designed pediatric trials. Despite regulatory constraints, early-life research can advance through safety-focused strategies, including food-based or low-dose approaches, validated models, and biomarker-driven assessments, supported by metabolomic and epigenetic profiling.
